# An Easy Method of Percentage Infant Feeding1Read before the Elmira Academy of Medicine, June, 1903.

**Published:** 1903-10

**Authors:** C. W. M. Brown

**Affiliations:** Elmira, N. Y.


### An Easy Method of Percentage Infant Feeding.[1]

By C. W. M. BROWN, M. D., Elmira, N. Y.

IF IT be true as has been claimed that 50 per cent. of American infants are fed by artificial methods, then there is urgent need that every general practitioner as well as pediatrists should be familiar with infant dietetics and the adaptation of suitable mixtures to the individual case. The history of the general consensus as to the best methods of artificial feeding of infants, reflects more rapid changes during the last few years than any other branch of medical practice. These changes have been brought about by a better understanding of the composition of human milk and a better knowledge of the bacteriology of milk and of the intestinal canal, as well as by a more thorough appreciation of the principles of feeding and nutrition. Biedert, thirty years ago, first gave an impetus to scientific milk examination and modification based upon chemical examination and made recommendation of mixtures of cream and milk, sweetened and made alkaline with lime water.

Ten years later, in 1882, the younger Meigs called attention to the low percentage of the proteids in human milk as well as the necessity for reducing the proteids in mixtures for artificial from cow's milk, and first worked out a formula that he believed corresponded with the analysis of human milk.

In 1886, Soxhlet proposed sterilisation of cows' milk by heating as the best safeguard against the intestinal diarrheas of bottle-fed infants. This had been practised for many years previously by Jacobi in our own country. For a time sterilisation became general all over the civilised world. Later, in 1890, Rotch made original contributions to percentage milk modification including the use of laboratory methods and prescription feeding. From this point all the recent advances have proceeded. The very fact that the laboratory methods were not available for general use made it necessary that others should devise ways and means by which we might approximate the advances suggested by Rotch. That so many methods and statements have been put forward concerning milk modifications suggested the lack of practicability inherent in most of them.

Methods of calculation or modifications have been proposed by Holt, Baner, Coit, Wescott, Taylor and Bartley and others, whereby various combinations more or less accurate of the percentage of fat, proteids, and sugar, could be determined in a mixture. These systems of modification were supposed to be

1. Read before the Elmira Academy of Medicine, June, 1903.

made available for the so-called home modification of milk in the absence of the milk laboratory; also for the guidance of those requiring accurate milk adjustment for the private case or for the large number of delicate infants in hospitals and other institutions. That the physician should be able to think in percentages to construct his own formulæ, reduce them to simple terms and transfer the modification of the mixtures to the caretaker, is absolutely necessary if he would do good work among the larger number of his feeding cases. The necessity, therefore, upon us for more general and careful study of infant feeding is becoming more and more urgent. We are altogether too prone to get ready-made foods and to fall back upon that strong hold of ignorance, that there are many children who "cannot take milk." The secret of successful feeding is to give a food which a child can digest. Age and weight are no guide in many cases. The key to success lies in putting the strength of the mixture down to so weak a point that a child can digest it, and then work it up to the point of tolerance.

The digestive organs of a young infant are exceedingly sensitive, easily deranged, and the simple functional derangement speedily becomes a gastric or intestinal catarrh. Hence the importance of paying attention to the food for the first month or two and of promptly and intelligently making necessary changes in the food to relieve the minor symptoms of indigestion. Feeble digestion or chronic indigestion are seldom due to inherited conditions, but in most cases are the result of previous bad feeding or bad nursing. The carefully obtained history is of great value in enabling one to judge of the condition and capabilities of the digestive organs of an individual child. A careful regulation of the milk percentages in intelligent hands will, in private practice, be successful in a great majority of cases. Success by this method may be extended in proportion to the accuracy of the diagnosis of the case, of the symptoms, and to the degree of error in the previous prescriptions employed. Cases that cannot be helped in this way are chiefly very young infants, and those in which the disorder is of long standing.

One of the secrets of the success obtained by many specialists in feeding children of weak digestion, lies in the fact that they boldly put the mixture down to very small percentages in spite of the fears of the mother that the child will not get sufficient food. That is the secret of the digestion of condensed milk. In a 1 to 12 dilution we have a food that contains but 1-8 of the amount of fat and 1-3 of the amount of proteid found in the normal breast milk. The fact that children do not do worse on these exceedingly weak milk mixtures is but one of many proofs that they commonly receive more than they require. If the doctor who is wedded to the use of condensed milk would not make his fresh milk mixtures four or five times as strong as his condensed milk mixtures, he would be much better satisfied with fresh milk.

The central thought in the newer modification which Holt aptly calls the "American method," is to consider the different elements of the food separately and to adapt their proportions to the child's digestion. It is based upon the percentage composition of woman's milk, and also recognises that there is a difference in the digestibility of cow's milk and woman's milk, particularly in the proteids. It aims to discover the proper proportions of fat, sugar and proteids and the best methods of increase in healthy infants. When difficulty exists in the digestion of milk it is usually with one of its constituents rather than with the milk as a whole, or at least, chiefly with one. In such a condition instead of stopping milk entirely, or reducing the proportions of all the constituents by further dilution, the proportions of that one which is causing the disturbance should be changed; in other words, percentage modification aims at something which is "definite, exact and at the same time flexible." It is perhaps a futile dream to hope that some day as a result of artificial selection and feeding, it will be possible to produce a cow that will yield milk that closely resembles in composition woman's milk. But as this has not yet been done, it is necessary to modify it, to use the technical phrase, in order to make it resemble woman's milk.

How, then, can modification be effected? It can be done in a milk laboratory or at home. The first involves considerable expense and may be impossible in the absence of a laboratory. The second or home modification although so simple in principle is, owing to the complexity with which it is already laid down in textbooks and in journal articles, often dreaded by the physician. Physicians revolt at doing an algebraic problem and considering an arithmetical sum at the bedside, and they also object to explain what they have been led to believe is a difficult and complicated process. On this account they either depend on the milk laboratory or solve the problem of infant feeding by telling the mother to get such and such a patent food and follow the directions. Many of these babies fed on patent foods go from bad to worse, although many thrive as they are in reality fed on crudely modified cow's milk with the addition of a food more or less useful, as the case may be.

The knowledge of home modification of milk would obviate this. This knowledge is necessary even when the conveniences of the laboratory can be obtained and where expense is not an object. For it is, I am sure, a fact that even with intelligent changing of the formulæ, laboratory modifications do not always agree. Infants are not machines and they are not all alike, and they will not always do what you expect of them. Laboratory milk is first divided into two parts by a centrifugal process,—one containing practically all the fat and the other part nearly free from it These two portions are then recombined with water, sugar of milk, and lime water in proportions to meet the formulæ required. In milk modifying at home the cream is obtained by natural separation, by standing.

In the *Boston Medical* and *Surgical Journal,* of March 25, 1899, Dr. Charles W. Townsend describes a method of milk modification which I have used for some years with satisfaction, and which seems to me to be the simplest of all, therefore I shall quote freely from his writings. He and others found by numerous tests that the upper fourth of milk of average quality after standing four or five hours contained approximately 10 per cent fat, 4 per cent. proteids, and 4 per cent. sugar. The percentage of albuminoids in this top milk is practically the same as in whole milk, that is 4 per cent. It will be seen that the relation between the fat and albuminoids is the same as in human milk, the fat being 2½ times as great, that is, 10 to 4, or 4 to 1.60 per cent. Top milk can be obtained by siphoning off the lower ¾, or more simply by pouring off the upper quarter. And it has been found that the cream obtained by these two methods is practically the same, varying according to Dr. Townsend's experiments only .17 of 1 per cent. The result being the same, the decanting process is preferable, it is much simpler and there is less difficulty in doing or having it done accurately and there is no siphon to be procured and kept clean. With a 10 per cent. cream it is evident that one ounce of cream in a 20 ounce mixture would give a percentage of 1-20 of 10 per cent. or one half of 1 per cent. of fat. In the same way the percentage of albuminoids would be 1-20 of 4 per cent. or .2 of 1 per cent. The percentage of sugar would be .2 of 1 per cent.

One more fact is needed before formulating a rule for human modification and that is that an even tablespoonful of sugar of milk weighs 3½ drams and each tablespoonful added to a 20 ounce mixture raises the percentage of sugar 2 per cent. It may be added that each tablespoonful of cane sugar which may sometimes be used, raises it 3 per cent. The rule to be remembered is this: each ounce of 10 per cent. cream in a 20 ounce mixture represents .50 of 1 per cent. of fat, .20 of albuminoids and .20 of sugar; and each even tablespoonful of sugar of milk represents 2 per cent., or of cane sugar 3 per cent. Thus, if we order top milk, 4 oz.; water, 15 oz.; lime water, 1 oz.; sugar of milk 2 tablespoonfuls, we are making a formula of fat 2 per cent., sugar, 4.8 per cent. and albumin .8 per cent. If we order 8 oz. of top milk, 11 oz. of water, 1 of lime water and 2½ tablespoonfuls of milk we are making a formula of fat 4, sugar 6.60 and so on. Of course, this extremely simple method gives, as I have said, a fixed relation between the fat and albumin, the fat being 2½ times as much as the albumin, the normal relation in human milk. If we wish to increase the albumin without the increase of fat it is necessary to add milk from which the cream has been removed, or whole milk, or top milk, containing less than 10 per cent. of fat.

By ordering whole milk which contains 4 per cent. of fat, 4 per cent. of proteids and 4 per cent. of sugar; the upper quarter which contains 10 per cent. of fat, 4 per cent. of proteids and 4 per cent. of sugar; the upper half of which contains 7 to 8 per cent. of fat, which for the purpose of calculation, may be called 8 per cent., 4 per cent. of proteids and 4 per cent. of sugar, or gravity cream which contains approximately 16 per cent. of fat, 4 per cent. of proteids, and 4 per cent. of sugar, and pursuing the same methods of division we can obtain almost any proportions of fat or proteids. The sugar can be raised to any desired amount by remembering the rule already given—namely, each level tablespoonful of sugar of milk added to a 20 oz. mixture raises the amount of sugar by 2 per cent., the amount of sugar in a mixture being the same as the amount of albuminoids. In digestive disturbances where the fat, sugar and albuminoids all have to be reduced to minute quantities the nutritive value of the mixture can be greatly increased without materially taxing the digestion by the addition of the raw white of egg. The white of an egg in a 20 oz. mixture adds half of 1 per cent. of albumin. One ounce of top milk, 18 oz. of water, 1 oz. of lime water and 1½ tablespoonfuls of sugar of milk give the formulæ: fat, .50 of 1 per cent.; sugar, 3.20 per cent.; albuminoids, .20 per cent., can be made much more nourishing by the addition of the whites of one or two eggs, and will be retained where the increase of the milk would not be. The proteids give most trouble in infantile digestion, therefore, their modification is most important.

Woman's milk contains twice as much lactalbumin, which is noncoagulable (0.9 to 0.5 per cent., Bartley) as casein, while cow's milk contains 2 or 3 times as much casein as lactalbumin (3. to 0.75, Bartley). Because of this, cow's milk coagulates as a fine dense mass in the infant's stomach, which is only lessened, but does not disappear, when the proteids are reduced to the same amount as in woman's milk. This necessitates making the proteids at beginning, feeding as low as 0.30 to 0.50 per cent. If this is done and the fat made as low as 1. to 1.50 per cent., the food strength may be gradually increased with slight risk that the milk will fail to agree, but if milk, especially the proteids, has been given too strong and continued until the child has a gastric or intestinal catarrh or both, then much time will be lost before food of proper strength and amount can be safely given.

Many other mixtures can be used in difficult cases, such as the use of whole or part whey mixtures, in which the casein has been coagulated with pepsin and strained out, with the addition of white of eggs and the use of cereal decoctions made from oatmeal, barley or rice. It is probably true that most children will do better after the seventh or eighth month with the use of a cereal decoction as a diluent for the milk, and in some cases they are undoubtedly of advantage in much younger infants.

Holt summarises the most important indications for varying the percentages as follows: if not gaining in weight and with no special signs of indigestion, increase proportions of all ingredients; if habitual colic, diminish the proteids; for frequent vomiting soon after feeding, reduce the quantity; for the regurgitation of sour masses of food, reduce the fat and sometimes the sugar; for obstinate constipation, increase both fat and proteids.

The criticism may be made that this system or method lacks accuracy. That it has the accuracy of the laboratory methods is not claimed, but all breast milk is not the same. All children would not thrive on the same breast. The most recent analysis shows that it is subject to rather wide variations in composition even when the nursling thrives in a normal way. This proves that the average infant can tolerate rather wide variations in its food without trouble and that extreme accuracy in the proportions of the constituents is not essential.

### CONCLUSIONS.

1. The modification of cow's milk for infant feeding may safely be done at home under the care of the mother, or nurse of ordinary intelligence, and is preferable to guess work.

2. It is based upon the known composition of mother's milk, and also upon the known dissimilarities between it and cow's milk, especially in the proteids.

3. The method of home modification and of calculating the percentages should and can be made extremely simple, and such modifications can be made sufficiently accurate and uniform.

4. The addition of cereals to the milk in the form of barley or oat-meal water is generally desirable after the seventh month, and is desirable before that age in some cases as an aid to the digestibility of the milk.

311 Baldwin Street.

